# Mental health in pregnant individuals during the COVID-19 pandemic based on a Swiss online survey

**DOI:** 10.1038/s41598-022-21881-2

**Published:** 2022-11-02

**Authors:** Guillaume Favre, Cléa Kunz, Simone Schwank, Ho-Fung Chung, Anda Petronela Radan, Luigi Raio, Mihaela Fluri, Ursula Winterfeld, David Baud, Léo Pomar

**Affiliations:** 1 Materno-Fetal and Obstetrics Research Unit, Department Woman-Mother-Child, Lausanne University Hospital, Lausanne, Switzerland; 2 Department of Clinical Science, Intervention and Technology (CLINTEC), Karolinska Institutet, Stockholm, Sweden; 3 Columbia University, College of Physicians and Surgeons, Center for Psychoanalytic Training and Research, New York, USA; 4 Department of Psychology, The University of Hong Kong, Hong Kong, China; 5 Department of Anthropology, The Chinese University of Hong Kong, Hong Kong, China; 6 Department of Obstetrics and Gynecology, University Hospital of Bern and University of Bern, Bern, Switzerland; 7 Swiss Teratogen Information Service, Clinical Pharmacology Service, Lausanne University Hospital and University of Lausanne, Lausanne, Switzerland; 8 School of Health Sciences (HESAV), University of Applied Sciences and Arts Western Switzerland, Lausanne, Switzerland; 9 Materno-Fetal & Obstetrics Research Unit, Department of Obstetrics and Gynecology, Centre Hospitalier Universitaire Vaudois (CHUV), 1011 Lausanne, Switzerland

**Keywords:** Epidemiology, Translational research, Risk factors, Public health

## Abstract

The aim of our study was to evaluate the mental health of pregnant individuals during the early COVID-19 pandemic and the potential factors associated. A Swiss online survey was proposed to individuals who gave birth during the pandemic period from March 2020. The Edinburgh Postnatal Depression Scale (EPDS), Generalized Anxiety Disorder 7 questions (GAD-7), and Impact Event Scale-Revised (IES-R) were evaluated and used to defined mental health impairment as a composite outcome. From October, 2020 to February, 2021, 736 participants responded. The anxiety GAD-7 score was moderate in 9.6% and severe in 2.0%. The EPDS was moderate in 21.5% and severe in 32.9%. The IES-R was moderate in 10.3% and severe in 3.9%. Mental health impairment was reported in 37.0%. The association between the risk of mental health impairment and foreign nationality was significant (OR = 1.48; 95%CI [1.06–2.05]) as well as fetal and pregnancy worries because of coronavirus (OR = 1.46; 95% CI [1.08–1.98]) and 1.65; 95% CI [1.22–2.24]). Adjusted ORs were significant for foreign nationality (aOR = 1.51; 95%CI [1.07–2.13]) and pregnancy worries because of coronavirus (aOR = 1.62; 95%CI [1.10–2.40]). Pregnant people and especially foreign national have a high risk of mental health impairment during the pandemic.

## Introduction

Following the first coronavirus disease 2019 (COVID-19) case in Switzerland in February 25, 2020, the situation rapidly escalated, with closure of all non-essential shops and services and the implementation of restrictions on social interactions^1^.

The measures undertaken to control viral spread have had multiple repercussions. While the Swiss confederation did not impose a complete lockdown as seen in many countries, the mental health of Swiss citizens was nonetheless severely impacted. Many factors contributed to this situation including potential financial instability, lack of social contact, drastic changes to routines, and health concerns amongst others. In the Swiss Corona Stress Study, while the proportion of participants reporting moderate depressive symptoms was 11.8% before the pandemic, this proportion rose to 24.7% after isolation measures were introduced. Severe depressive symptoms were reported in 2.4% and 9.1%, respectively. Furthermore, 57% of participants felt more anxious during the pseudo-lockdown^2^. Of note, a longitudinal study in Argentina also reported that the pandemic had contributed to a deterioration of mental health, leading to increased susceptibility to depressive and anxiety symptoms among pregnant individuals^3^.

Pregnant individuals are particularly vulnerable to mental health disorders due to life changes, stress and hormonal adaptations. Furthermore, depression and anxiety are frequently underdiagnosed during pregnancy^4^. Additionally, concerns regarding medication safety rise during pregnancy and many patients discontinue their antidepressant or anxiolytic medications, increasing the risk of relapse^5^. In Switzerland, one in six pregnant people require mental health services each year, with depression being the most prevalent perinatal mental health related diagnosis^6^. Maternal distress, including depression, anxiety, and stress, are critical as they increase the risk of miscarriage^7^. Low birth weight, as well as preterm birth, are also increased in pregnancies affected by maternal mental health disorders^8,9^. It can also impact the long-term health of children with a higher rate of severe psychological diagnoses, ranging from autism to schizophrenia^10,11^.

The COVID-19 pandemic intensified the mental strain of pregnancy due to legitimate concerns consisting in an increased vulnerability to a severe COVID-19 infection compared to non-pregnant individuals of the same age, along with the consequence of their fetus^12^. Depression and anxiety amongst pregnant people have substantially increased during this outbreak worldwide^13–15^. Alongside from general fears arising during pregnancy, health concerns for pregnant individuals following a COVID-19 infection have emerged^16^. It has been established that pregnant people are at higher risk of infection and progression to a severe form of COVID-19, with a higher risk of intensive care unit admission, mechanical ventilation, and death^12,17,18^. Preterm birth is increased in pregnancies to mothers that tested positive for the virus, and potential dramatic neonatal issues have also been reported in the literature^19,20^. Fear of COVID-19 infection led some pregnant individuals to not adhere to routine antenatal surveillance deepening their anxiety state and indirectly increasing the rate of stillbirth^21^. Additionally, social isolation, poor access to support, and economic uncertainty have amplified depressive symptoms. Previous studies have reported that the first wave of the pandemic may have had a severe impact on pregnant individuals’ mental health in Switzerland and suggested to set up specific prevention and support strategies for this high risk group^22^. A recent study in Sweden reported high prevalence of depression and anxiety in pregnancy during the COVID-19 highlighting the need to prevent and support this population even late in the pandemic. So far, no study so far has evaluated the mental health of pregnant people later in the pandemic in Switzerland.

The aim of our study was to evaluate the mental health status of pregnant individuals during the late COVID-19 pandemic in Switzerland and to explore the potential factors associated with altered mental health outcomes.

## Methods

### Design and data collection

The study was conducted from October 28, 2020 to February 23, 2021. Participants were recruited with an announcement on Lausanne University Hospital (CHUV) and Bern University Hospital (Inselspital) public social media platforms (Facebook). The announcement included the link to the online survey (powered by Qualtrics, Provo, UT). After voluntarily signing an informed consent form, participants accessed the online questionnaire. This form was available in French, German, and Italian, and required approximately 20 min to complete. Anonymous data was self-reported by individuals who consented to participate in the study. Data was collected in accordance with the General Data Protection Regulation (GDPR 2016/679) on privacy in the European Union and the European Economic Area. Institutional review board (IRB) approval from Lausanne University Hospital was obtained. During the study period, measures to fight coronavirus in Switzerland have varied over time and included compulsory masks in every closed space including transportations and public spaces, prohibition of classroom courses, private events with more than 5 to 10 persons and cultural/sports manifestation. Nightclubs/discos and bars were also closed. People travelling to Switzerland from foreign countries were quarantined. Measures have been reinforced from December2020–January 2021 with the closing of every non-essential shops and the recommendation to avoid unnecessary private gatherings. People were asked to stay at home^23^.

### Participants

Individuals who were pregnant at the time of the questionnaire were eligible. Exclusion criteria were pregnant people who were not of legal age (< 18 years old), not able or willing to consent to participate in the study or who did not speak any of the survey languages.

### Co-variates

Maternal age was divided into categories: ≤ 25 years (y), 26 to 30 y, 31 to 35 y, 35 to 40 y, and > 40 y. Baseline characteristics of participants were also collected as follows: ethnicity, nationality, marital status, work hours per week, family income per year, and educational level. Foreign nationality was defined as a participant who is not Swiss. Amount of work hours per weeks (h/w) were divided into categories: less than 40 h/w, 40 h/w, and more than 40 h/w. Higher education was reported as having a bachelor’s degree or more. High family income was considered as 90,000 Swiss francs (CHF) or more per year according to the mean household incomes in Switzerland^24^.

### Primary outcome: COVID-19 concerns, depressive symptoms, anxiety and impact of events

The questionnaire was composed of several blocks of questions, based on participants’ concerns about the pandemic as well as three mental health evaluation scales: the Edinburgh Postnatal Depression Scale (EPDS), the Generalized Anxiety Disorder 7 questions (GAD-7), and the Impact Event Scale–Revised (IES-R).

### COVID-19 pandemic concerns

Questions about the COVID-19 pandemic were specifically designed for this study, based on authors’ expertise from clinical experience and patients feedbacks at the beginning of the pandemic. Questions assessed participants’ concerns about their health, anxiety, pregnancy, fetus, as well as the concerns from family members about the respondent being pregnant during the pandemic. The 9 questions were (1) Are you concerned about your health status because of the Coronavirus?, (2) Are you anxious about the coronavirus?, (3) Are you worried about being pregnant during the coronavirus period?, (4) Are you worried about your fetus in relation to the coronavirus?, (5) Are you worried about your fetus in relation to the coronavirus?, (6) Are your family members concerned of you being pregnant during the coronavirus?, (7) How does the media information impact your worries about the coronavirus?, (8) Is the health care professional’s information useful to reduce your questions and concerns regarding the coronavirus ?, (9) Would you like more information regarding the coronavirus related to pregnancy? For the first 6 questions, answers were evaluated on a scale of 0 to 10, 0 being “not anxious at all” and 10 “very anxious”. For the last three questions, answers were evaluated on a scale of 0 to 10, 0 being “not at all” and 10 “extremely”. Answers were pooled into 5 category scores: 0–1, 2–4, 5–7, and 8–10 for visual interpretation.

### General anxiety

General anxiety was evaluated by the generalized anxiety disorder 7 questions (GAD-7); a 7 item self-reported anxiety scale assessing the severity of general anxiety over the last 2 weeks. The scale involves indicating the frequency at which the patient is bothered by specific situations using a four-point Likert scale, ranging from not at all to nearly every day (score 0 to 3, respectively), giving a total score out of 21. Total GAD-7 scores were classified into minimal (0–4), mild (5–9), moderate (10–14), and severe anxiety (15–21)^25^.

### Depressive symptoms

Depressive symptoms were assessed using the Edinburgh Postnatal Depression Scale (EPDS), a self-reported questionnaire composed of 10 questions. For each question, four suggestions are present, each corresponding to a score from 0 to 3, rating the intensity of depressive symptoms over the last 7 days for a final score out of 30. A score of ≥ 13 was considered representative of major depressive symptoms^26^.

### Impact of events

The impact event scale-revised (IES-R) is a 22-item self-reported measure of subjective distress caused by traumatic events. Patients are asked to identify a specific stressful life event and indicate how much they were distressed or bothered by it during the past seven days by each difficulty listed. Total score ranged from 0 to 88 and were categorized into mild (0–39), moderate (40–55), and severe (56–88) symptoms^27^.

### Secondary outcome: mental health impairment

Mental health impairment was a composite outcome built for this study, defined as at least one of the following conditions: (i) GAD-7 score ≥ 10 (ii) EPDS score ≥ 13 or (iii) IES-R score ≥ 40. This was not a validated instrument and it was only used to analyze potential associated cofactors.

### Statistical analysis

A descriptive analysis was performed regarding participants’ basic characteristics, COVID-19 situation concerns, and mental health scores reporting absolute numbers of participants, proportions in percentages, means and 95% confidence intervals.

A case control analysis was performed, assessing the association between potential risk factors and risk of mental health impairment. Risk factors assessed were foreign nationality, maternal age > 35 (as it represents a known risk factor for pregnant individuals)^28,29^, married status, working hours more than 40 h/w, high educational level, high family income, fetal worries because of COVID-19 score ≥ 5/10, and pregnancy worries because of COVID-19 score ≥ 5/10. Univariate logistic regression was performed, reporting crude Odds Ratios (OR). A multivariate logistic regression analysis was performed. All potential risk factors were considered in the model. A p value less than 0.05 was considered as statistically significant. Statistical analyses were performed using Stata 14 (StataCorp. 2015. Stata Statistical Software: Release 14. College Station, TX: StataCorp LP).

## Results

From October 28, 2020, to February 23, 2021, 736 participants that gave birth during the pandemic period responded to the questionnaire and were included. Mean maternal age was 32 years (interquartile range 30–35). Most participants (n = 559; 76.0%) were married or were cohabitating with their partner and 72.3% (n = 532) respondents were Swiss. The percentage of participants that were working more than 40 h a week was 28.1% (n = 207), had a family income of more than 90,000 CHF per year was 59.1% (n = 435), and had a higher education with a bachelor’s degree or more was 78.0% (n = 574) (Table 1).

**Table 1:** Basic characteristics of participants.

	Patients n = 736		
	n	%	IQR
**Maternal age in years (y)**			
18–25 y	24	3.3	2.1–4.8
26–30 y	219	29.8	26.5–33.2
31–35 y	350	47.6	43.9–51.2
36–40 y	117	15.9	13.3–18.7
> 40 y	26	3.5	2.3–5.1

	n	%	95% CI
**Marital/relationship status**			
Single	57	7.7	5.9–9.9
Married	421	57.2	53.5–60.8
Cohabitation	138	18.8	16.0–21.8
In relationship	118	16.0	13.5–18.9
Divorced	2	0.3	0.0–1.0
**Nationality**			
Swiss	532	72.3	68.9–75.5
French	122	16.6	14.0–19.5
Belgium	31	4.2	2.9–5.9
German	13	1.8	0.9–3.0
Italian	8	1.1	0.5–2.1
Other	30	4.1	2.8–5.8
**Working hours per week**			
Less than 40 h a week	419	56.9	53.3–60.5
40 h a week	110	14.9	12.4–17.7
More than 40 h a week	207	28.1	24.9–31.5
**Family income per year (CHF-Swiss francs)**			
Less than 90,000 CHF	216	29.3	26.1–32.8
Around 90,000 CHF	71	9.6	7.6–12.0
More than 90,000 CHF	435	59.1	55.5–62.7
Unknown	14	1.9	1.0–3.2
**Higher educational level**			
No scholar education	4	0.5	0.1–1.4
Secondary school 12–15 years	19	2.6	1.6–4.0
Secondary school 15–18 years	16	2.2	1.2–3.5
Diploma	120	16.3	13.7–19.2
Bachelor degree	167	22.7	19.7–25.9
Master degree	312	42.4	38.8–46.1
Doctorate degree/PhD	95	12.9	10.6–15.5
Unknown	3	0.4	0.1–1.2

### Primary outcome

#### COVID-19 pandemic concerns

**Figure 1:**
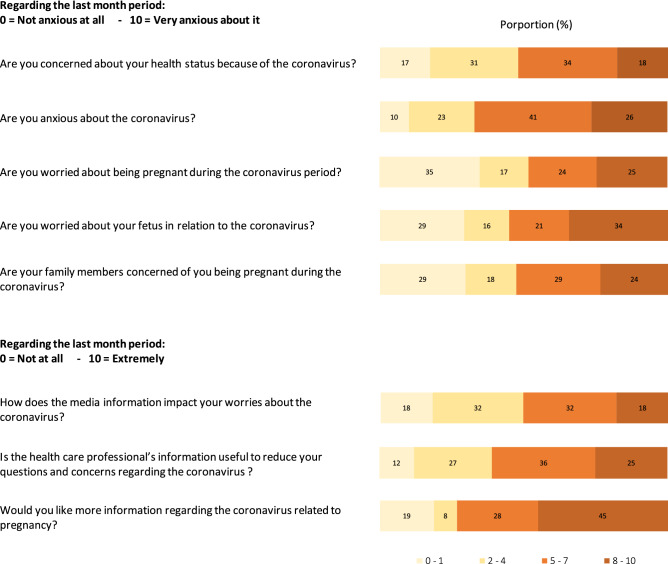
COVID-19 concerns of participants.

Participants’ concerns regarding the COVID-19 pandemic are presented in Fig. 1.

18 to 34% of individuals (131 to 253/736) reported being very anxious about COVID-19-related concerns with a score of ≥ 8 out of 10. A proportion of 40% (296/736) of participants reported that they were concerned about their health status because of coronavirus with a score ≥ 5/10 (mean score 4.6; 95%CI 4.4–4.8). The major concern was the global coronavirus situation with an anxiety score ≥ 5/10 in 67% (493/736—mean score 5.5; 95%CI 5.3–5.7). Concerns about being pregnant during the coronavirus period and worries about their fetus in relation to the coronavirus was reported with a score ≥ 5/10 in 40% (295/736—mean score 4.25; 95%CI 4.0–4.5) and 49% (364/736—mean score 5.0: 95%CI 4.7–5.3) respectively. A total of 53% (388/736) reported one of their family member being worried about their pregnancy in the COVID-19 context with a score ≥ 5/10 (mean score 4.5; 95%CI 4.2–4.7). Half of the participants concerns were impacted by the media (50.4%, 371/736—mean score 4.4; 95%CI 4.2–4.7) with a score ≥ 5/10. The majority of individuals reported that information from health care professionals was useful in reducing anxiety associated with pregnancy during the coronavirus pandemic, with a score ≥ 5/10 in 61% (448/736—mean score 5.2; 95%CI 5.0–5.4). The majority expressed their interest in receiving more information on SARS-CoV.2 and pregnancy with a score ≥ 5/10 in 73% (538/736—mean score 6.1; 95%CI 5.9–6.4).

#### General anxiety, depressive symptoms, and impact of events

The mental health score assessment of respondents is reported in Table 2. Anxiety disorder was evaluated by the GAD-7 as mild in 38.3% (n = 282), moderate in 9.6% (n = 71), severe in 2.0% (n = 15), and missing in 0.3% (n = 2). Depressive symptoms were evaluated by the EPDS as minimal in 45.7% (n = 336), moderate in 21.5% (n = 158), and severe in 32.9% (n = 242). The IES-R score that assessed distress caused by traumatic events was reported as mild in 85.7% (n = 631), moderate in 10.3% (n = 76), severe in 3.9% (n = 29), and missing in 14.3% (n = 105).

**Table 2:** Mental health assessment using GAD-7, EPDS and IES-R among participants.

		Patients n = 736		95% CI
		n	%	
GAD-7	Minimal (0–4)	366	49.7	46.1–53.4
	Mild (5–9)	282	38.3	34.8–41.9
	Moderate (10–14)	71	9.6	7.6–12.0
	Severe (15–21)	15	2.0	1.1–3.3
	Missing	2	0.3	0.0–1.0
EPDS	Minimal (< 10)	336	45.7	42.0–49.3
	Moderate (10–12)	158	21.5	18.6–24.6
	Severe (≥ 13)	242	32.9	29.5–36.4
IES-R	Mild (< 40)	631	85.7	83.0–88.2
	Moderate (40–55)	76	10.3	8.2–12.8
	Severe (> 55)	29	3.9	2.7–5.6
	Missing	105	14.3	11.8–17.0

### Secondary outcome

#### Mental health impairment

A total of 272 (37.0%) participants were identified to have a mental health impairment. Baseline characteristics of patients, according to mental health impairment status, are described in supplementary materials—Table S1. The association between the risk of mental health impairment and foreign nationality was significant with an OR of 1.48 (95%CI [1.06–2.05]; p = 0.021) as well as fetal and pregnancy worries because of coronavirus with a crude OR of 1.46 (95% CI [1.08–1.98]; p = 0.014) and 1.65 (95% CI [1.22–2.24]; p = 0.001). All other covariates, maternal age ≥ 35 years, marital status, working more than 40 h a week, high educational level, and low family income were not significant. Adjusted ORs were only significant for foreign nationality (aOR = 1.51; 95%CI [1.07–2.13]; p = 0.020) and pregnancy worries because of coronavirus (aOR = 1.62; 95%CI [1.10–2.40]; p = 0.016). Other covariates adjusted ORs were not significant (Table 3).

**Table 3:** Associations between mental health impairment and baseline characteristics as potential risk factors using a univariate and a multivariate logistic regression model.

	Patients without mental health impairment			Patients with mental health impairment			OR	95% CI	p	aOR	95% CI	p
	n = 464			n = 272								
	n	%	95% CI	n	%	95% CI						
Foreign nationality	115	24.8	20.9–29.0	89	32.7	27.2–38.6	1.48	1.06–2.05	0.021	1.51	1.07–2.13	0.020
Maternal age > 35 y	134	28.9	24.8–33.2	64	23.5	18.6–29.0	0.76	0.54–1.07	0.115	0.78	0.55–1.11	0.168
Married/cohabitation	358	77.2	73.1–80.9	201	73.9	68.3–79.0	0.84	0.59–1.19	0.318	0.85	0.59–1.21	0.355
Working hours more than 40 h/w	123	26.5	22.5–30.8	84	30.9	25.4–36.7	1.24	0.89–1.72	0.203	1.30	0.93–1.83	0.123
High educational level (bachelor or more)	368	79.3	75.3–82.9	209	76.8	71.4–81.7	0.87	0.60–1.24	0.432	0.80	0.55–1.16	0.239
High family income (more than 90,000 CHF/year)	335	72.2	67.9–76.2	185	68.0	62.1–73.5	0.82	0.59–1.13	0.229	0.91	0.65–1.28	0.597
Fetal worries because of COVID-19	240	51.7	47.1–56.4	166	61.0	55.0–66.9	1.46	1.08–1.98	0.014	1.11	0.75–1.65	0.606
Pregnancy worries because of COVID-19	203	43.8	39.2–48.4	153	56.3	50.1–62.2	1.65	1.22–2.24	0.001	1.62	1.10–2.40	0.016

y: years; h/w: hours per week; OR: crude odd ratio; aOR: adjusted OR; Adjustment with all cofactors listed in the table.

## Discussion

This study reports that pregnant individuals seemed to have been particularly impacted by the COVID-19 pandemic with almost one third of participants experiencing severe symptoms of depression. In addition, 11.6% of participants experienced moderate to severe symptoms of anxiety with a GAD-7 score of ≥ 10 and 14.2% reported a moderate or severe impact caused by traumatic events. During the pandemic, 37.0% of pregnant individuals reported as having a mental health impairment.

The impact of the COVID-19 pandemic on mental health is potentially serious with most participants stating that they were concerned about their health, their pregnancy, and their unborn child due to coronavirus and two thirds of the participants reporting anxiety surrounding the pandemic situation.

Information given by health care providers was perceived as reassuring and the participants expressed their interest in receiving more information. Persons experiencing worries about their pregnancy because of COVID-19 were at higher risk of mental health impairment highlighting that pregnancy represents a relevant risk factor itself. Pregnant individuals identified as foreign nationals were also significantly more at risk for mental health impairment suggesting that possible interventions should target this population.

Our results align with current literature. International studies have found that severe depressive symptoms affected 13.9% and moderate to severe anxiety affected 16.7% of people during and after pregnancy^30^. Our results suggest a much higher proportion of mothers experiencing symptoms of depression in Switzerland and less generalized anxiety. Switzerland has not imposed a strict lockdown unlike many other countries, suggesting that the lack of social interactions may not be the main cause of perinatal depression. Our results, however, suggest a significant association between mental health impairment and foreign nationality during the pandemic. Switzerland is composed of approximately 25% foreign nationals in 2020^31^. Border closures could have mimicked lock down conditions on a larger scale, limiting foreign nationals from returning to their family for support during this critical life event. While the prevalence of perinatal maternal disorders is on the rise, especially during this global pandemic, studies have suggested that psychiatric supports for these individuals are deficient in Switzerland, and highlights in particular the lack of guidelines, routine evaluations, as well as progress in the field^32^. These results are comparable to already published data with a similar methodology, reporting higher mental health impairment according to Khoury et al. in a similar high educated pregnant population. Lack of social support was associated as a risk factor for exacerbating mental health symptoms mirroring our results regarding foreign nationality^33^. Low socioeconomic status (SES) appears to have played a role as a risk factor for impaired mental health during the pandemic period, as reported in a study of a non-pregnant population in Switzerland. However, low SES was also associated with fear of employment loss due to the crisis, which can be compared with the situation of pregnant people, who are more vulnerable to the risk of job loss. Pregnancy can be considered as a constraint because of the employer's obligation to adapt the job and working hours of the person concerned. The health crisis has increased this precariousness because the pregnant person is at risk of a severe form of COVID-19 and the employer is responsible for the safety of its employee^34^. Dismissal is therefore a legitimate fear for the pregnant person during this period and probably plays an additional role thus explaining that SES could not be as protective in pregnant individuals than in the general population.

Our study has several limitations including a selection bias as the survey was voluntary and only accessible via the online maternity social platform. Participants with a higher SES and educational background who were concerned by the current situation may have had easier access to the survey, which is consistent with the basic characteristics of our participants. The mental health assessment was limited to score scales not evaluating the medical history of mental health disorders or any other medical conditions that could have added to participant concerns. The COVID-19 concerns questions were built for the study only to try to describe maternal concerns but are not a validated tool. Similarly, the mental health impairment status outcome was constructed for the study based on 3 validated scores as previously described. This composite outcome is not a validated score but allowed us to extend the analysis to potential covariates associated with this outcome. Additionally, the study did not collect information about maternal disease and pregnancy conditions arising during pregnancy that have been reported to play a role on mental health^35^. Finally, these results were collected at a time where the pandemic had led to the implementation of strong protective social measures. The results do not necessarily apply to the current situation due to various reasons including current less severe SARS-CoV 2 variants and massive vaccination campaigns.

This study, however, provides a snapshot of how the mental health status of pregnant individuals during a difficult situation may have been affected and provides us with clues on how to better manage such worries in an already stressed context that represents pregnancy.

The study suggests that pregnant persons were at risk of mental health disorder even at distance of the first wave of COVID-19 and first scientific evidences that came a few months after the beginning of the pandemic. This emphasize that clinicians should be aware of pregnant individuals during these periods and give particular attention to foreign individuals even with a high educational background or a high income. These results could be useful for any resurgence of a new mutation of SARS-CoV-2 or potential new emerging pathogens such as monkey pox-virus or still unknown future pathogens^36^.

Further studies are needed, especially later in the pandemic, to distinguish the impact of social restrictions from that of the pandemic itself, on psychological distress among pregnant individuals.

## Conclusion

This study suggests that pregnant people have a high risk of mental health impairment during the pandemic. Pregnant individuals should therefore be better informed about the impacts of the pandemic on pregnancy, and this should be a key focus of health care professionals. Emphasis should be placed on vulnerable populations, for example foreign nationals regardless of SES or educational status.

## Supplementary Information

Supplementary Table S1.

## Data Availability

The datasets generated and/or analyzed during the current study are not publicly available. Participants have signed an informed consent stating that data are not publicly available but only to the dedicated research team or collaborators of the research team for additional research work only. Data can be available from the corresponding author on reasonable request under joint research agreement.
