# Supplementary material for: Mental health in pregnant individuals during the COVID-19 pandemic based on a Swiss online survey
Source: Sci Rep. 2022 Nov 2;12:18448. doi: 10.1038/s41598-022-21881-2 (PMC9628478; doi:10.1038/s41598-022-21881-2)
Supplement: Supplementary file 1 — Supplementary Table S1. [file 41598_2022_21881_MOESM1_ESM.pdf]

Supplementary materials

MENTAL HEALTH IN PREGNANT INDIVIDUALS DURING THE COVID-19 PANDEMIC BASED ON A SWISS ONLINE SURVEY

Guillaume Favre, Cléa Kunz, Simone Schwank, Ho-Fung Chung, Anda Petronela Radan, Luigi Raio, Mihaela Fluri, Ursula Winterfeld, David Baud & Leo Pomar

Table S1: Baseline characteristics between participants without and with mental health impairment

	Patient **without** mental health impairment n = 464					Patient **with** mental health impairment n = 272				
	**n**	**%**	**IQR**			**n**	**%**	**IQR**		
**Maternal age (years)**										
- 18-25	12	2.6	1.3	-	4.5	12	4.4	2.3	-	7.6
- 26-30	138	29.7	25.6	-	34.1	81	29.8	24.4	-	35.6
- 31-35	216	46.6	41.9	-	51.2	134	49.3	43.2	-	55.4
- 36-40	81	17.5	14.1	-	21.2	36	13.2	9.4	-	17.8
- >40	17	3.7	2.1	-	5.8	9	3.3	1.5	-	6.2
	**n**	**%**	**95%CI**			**n**	**%**	**95%CI**		
**Marital / Relationship status**										
- Single	34	7.3	5.1	-	10.1	23	8.5	5.4	-	12.4
- Married	280	60.3	55.7	-	64.8	141	51.8	45.7	-	57.9
- Cohabitation	78	16.8	13.5	-	20.5	60	22.1	17.3	-	27.5
- In relationship	70	15.1	12.0	-	18.7	48	17.6	13.3	-	22.7
- Divorced	2	0.4	0.1	-	1.5	0	0.0	0.0	-	1.3
**Nationality**										
- Swiss	349	75.2	71.0	-	79.1	183	67.3	61.4	-	72.8
- French	72	15.5	12.3	-	19.1	50	18.4	14.0	-	23.5
- Belgium	12	2.6	1.3	-	4.5	19	7.0	4.3	-	10.7
- German	9	1.9	0.9	-	3.7	4	1.5	0.4	-	3.7
- Italian	2	0.4	0.1	-	1.5	6	2.2	0.8	-	4.7
- Other	20	4.3	2.7	-	6.6	10	3.7	1.8	-	6.7
**Working hours per week**										
- Less than 40 hours a week	272	58.6	54.0	-	63.1	147	54.0	47.9	-	60.1
- 40 hours a week	69	14.9	11.8	-	18.4	41	15.1	11.0	-	19.9
- More than 40 hours a week	123	26.5	22.5	-	30.8	84	30.9	25.4	-	36.7
**Family income per year (CHF - Swiss francs)**										
- Less than 90.000 CHF	129	27.8	23.8	-	32.1	87	32.0	26.5	-	37.9
- Around 90.000 CHF	39	8.4	6.0	-	11.3	32	11.8	8.2	-	16.2
- More than 90.000 CHF	286	61.6	57.0	-	66.1	149	54.8	48.7	-	60.8
- Unknown	10	2.2	1.0	-	3.9	4	1.5	0.4	-	3.7
**Higher educational level**										
- No scholar education	1	0.2	0.0	-	1.2	3	1.1	0.2	-	3.2
- Secondary school 12-15 years old	15	3.2	1.8	-	5.3	4	1.5	0.4	-	3.7
- Secondary school 15-18 years old	10	2.2	1.0	-	3.9	6	2.2	0.8	-	4.7
- Diploma	70	15.1	12.0	-	18.7	50	18.4	14.0	-	23.5
- Bachelor degree	105	22.6	18.9	-	26.7	62	22.8	17.9	-	28.2
- Master degree	201	43.3	38.8	-	48.0	111	40.8	34.9	-	46.9
- Doctorate degree / PhD	60	12.9	10.0	-	16.3	35	12.9	9.1	-	17.4
- Unknown	2	0.4	0.1	-	1.5	1	0.4	0.0	-	2.0
